# Supplementary material for: The interactome of intact mitochondria by cross-linking mass spectrometry provides evidence for coexisting respiratory supercomplexes
Source: Mol Cell Proteomics. 2017 Dec 8;17(2):216–32. doi: 10.1074/mcp.RA117.000470 (PMC5795388; doi:10.1074/mcp.RA117.000470)
Supplement: Supplemental Data [file supp_RA117.000470_133922_0_supp_23978_fzffwf.zip › spectra_annotation/mito_DR_spectra_annotation/225-1-9-1-11-1.pdf]

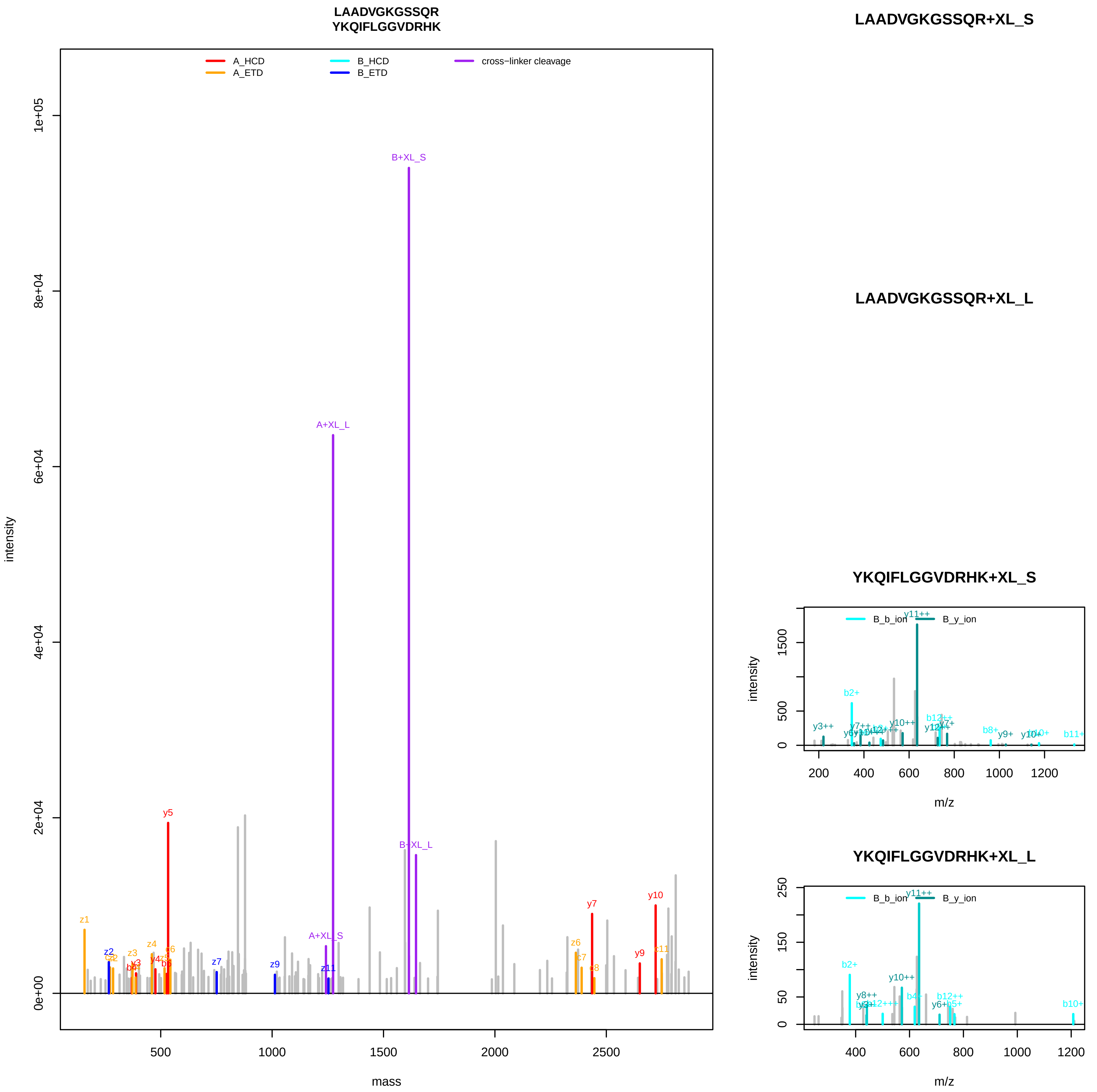

LAADVGKGSSQR
YKQIFLGGVDRHK
A_HCD
A_ETD
B_HCD
B_ETD
cross−linker cleavage
intensity
mass
0e+00
2e+04
4e+04
6e+04
8e+04
1e+05
500
1000
1500
2000
2500
B+XL_S
A+XL_L
B+XL_L
A+XL_S
y5
y7
y9
y10
z1
z2
z3
z4
z6
z7
z9
z11
c7
c8
c11
LAADVGKGSSQR+XL_S
LAADVGKGSSQR+XL_L
YKQIFLGGVDRHK+XL_S
B_b_ion
B_y_ion
intensity
m/z
y11++
b2+
y3++
y10++
b8+
y9+
y10+
b11+
YKQIFLGGVDRHK+XL_L
B_b_ion
B_y_ion
intensity
m/z
y11++
b2+
y10++
b10+
